# The biogenesis, function and clinical significance of circular RNAs in breast cancer

**DOI:** 10.20892/j.issn.2095-3941.2020.0485

**Published:** 2022-01-15

**Authors:** Yan Zeng, Yutian Zou, Guanfeng Gao, Shaoquan Zheng, Song Wu, Xiaoming Xie, Hailin Tang

**Affiliations:** 1 Sun Yat-sen University Cancer Center; State Key Laboratory of Oncology in South China; Collaborative Innovation Center for Cancer Medicine, Guangzhou 510060, China

**Keywords:** Breast cancer, circular RNAs, carcinogenesis, tumor biomarker, targeted therapy

## Abstract

Circular RNAs (circRNAs) are noncoding RNAs that form covalently closed loop structures. CircRNAs are dysregulated in cancer and play key roles in tumorigenesis, diagnosis, and tumor therapy. CircRNAs function as competing endogenous RNAs or microRNA sponges that regulate transcription and splicing, binding to proteins, and translation. CircRNAs may serve as novel biomarkers for cancer diagnosis, and they show potential as therapeutic targets in cancers including breast cancer (BC). In women, BC is the most common malignant tumor worldwide and the second leading cause of cancer death. Although evidence indicates that circRNAs play a critical role in BC, the mechanisms regulating the function of circRNAs in BC remain poorly understood. Here, we provide literature review aiming to clarify the role of circRNAs in BC and summarize the latest research. We provide a systematic overview of the biogenesis and biological functions of circRNAs, elaborate on the functional roles of circRNAs in BC, and highlight the value of circRNAs as diagnostic and therapeutic targets in BC.

## Introduction

Breast cancer (BC) is the malignant tumor type with the highest incidence among women worldwide and the second leading cause of cancer-related death among women. According to the 2019 statistics from the American Cancer Society, there were 268,600 new cases of BC in women worldwide, accounting for 30% of all new cancer diagnoses in women, and 41,760 deaths, accounting for 15% of cancer deaths among women^1,2^. Despite the availability of treatments such as surgery^3^, chemotherapy^4^, and targeted therapy^5^, BC has not yet been fully and effectively controlled^6,7^. BC has a high mortality rate partly attributed to the rates of recurrence and metastasis. In the United States, each year, more than 150,000 women receive a diagnosis of metastatic BC, and nearly 41,000 deaths from BC occur, virtually all of which are due to metastatic disease^6,8^. The overall 5-year BC survival rate for patients diagnosed between 2009 and 2015 is 98% for stage I and 27% for stage IV^1^. There is an urgent need to develop new BC therapies and to identify novel diagnostic markers and therapeutic targets for BC.

In recent years, after substantial research on microRNAs (miRNAs) and long non-coding RNAs (lncRNAs), circular RNAs (circRNAs) have become a new focus of studies in the field of noncoding RNA. CircRNAs are noncoding RNAs characterized by single-stranded, covalently closed circular transcripts that lack 5′ caps and 3′ poly(A) tails. These features make circRNAs resistant to digestion by ribonucleases, such as RNase R and exonuclease, thus resulting in longer half-lives than those of linear mRNAs^9^. CircRNAs were first discovered as viroids in RNA viruses by electron microscopy in 1976^10^ and were found to be endogenous RNA splicing products in eukaryotes in 1979^11^. In subsequent decades, circRNAs were considered to be a result of splicing errors, and only the testis-specific circRNA from the sex-determining region Y (*SRY*) gene was considered to have a potential function^12^. However, in the 21st century, the development of RNA sequencing (RNA-seq) technologies and bioinformatics led to the discovery that circRNAs are widespread in eukaryotic cells and are involved in the biogenesis and development of various diseases, including nervous system disorders^13^, cardiovascular disorders^14^, and cancer^15–18^. For example, the cancer landscape of circRNAs in brain, lung, thyroid, breast, and bladder cancers was determined through an exome capture RNA-seq protocol, which detected and characterized circRNAs across more than 2000 cancer samples^19^. CircRNAs play critical roles in carcinogenesis, metastasis, and resistance to therapy^18,20^. The stability and tissue specificity of circRNAs in exosomes or body fluids^21,22^ suggest that circRNAs may serve as reliable tumor biomarkers for diagnosis or prognostication, as well as potential therapeutic targets in various cancers including BC^23,24^.

A systematic understanding of the functions and mechanisms underlying the roles of circRNAs in BC tumorigenesis and development may contribute to the discovery of novel diagnostic methods and effective therapies. Here, we provide an overview of the biogenesis and biological functions of circRNAs, elaborate on the functional roles of circRNAs in BC, and highlight the value of circRNAs as diagnostic factors and therapeutic targets in BC.

## The biogenesis of circRNAs

**Figure 1:**
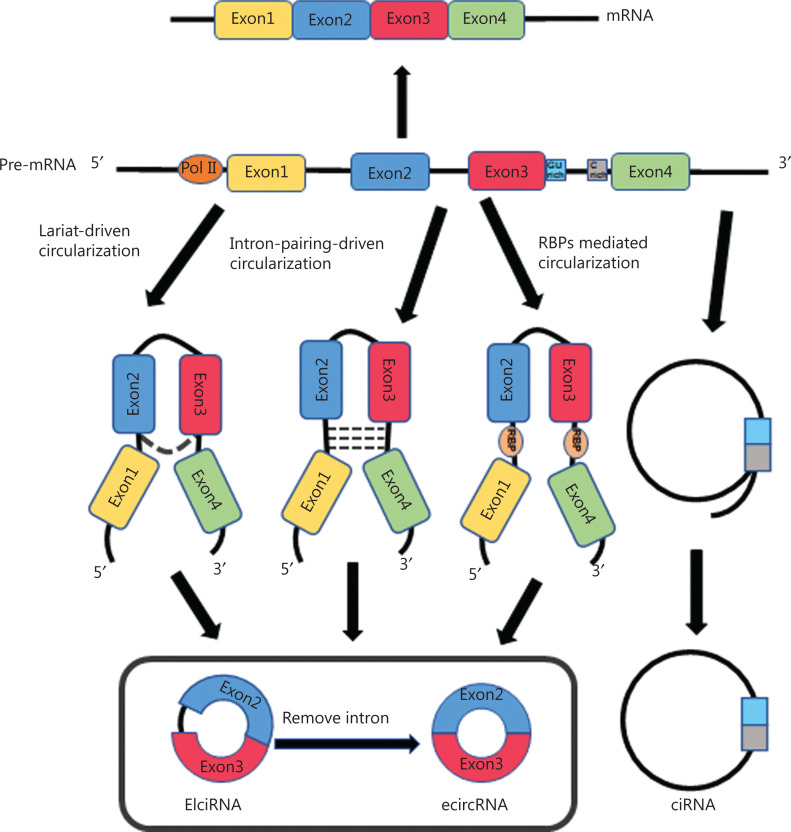
The biogenesis of circRNAs. ecircRNA is formed in 3 ways: lariat-driven circularization, intron-pairing-driven circularization, and RBP mediated circularization. EIciRNA is composed of exons and retained introns. ciRNAs, containing a 7 nt GU-rich element adjacent to the 5′ splice site and an 11 nt C-rich element adjacent to the branch point site, are formed during splicing.

CircRNAs are formed through pre-mRNA back-splicing, which connects a downstream splice donor site (5′ splice site) to an upstream acceptor splice site (3′ splice site), and transcription by RNA polymerase II (RNA pol II)^25^. Different circRNAs can be formed from the same sequences through alternative back-splicing. Despite years of intense research efforts, the exact mechanism underlying circRNA formation is not completely understood. According to their composition and cycling mechanisms, circRNAs are divided into 3 groups: exonic circRNAs (ecircRNAs)^26,27^, exon-intron circRNAs (ElciRNAs)^28^, and circular intronic RNAs (ciRNAs)^29^. ecircRNAs are predominantly localized to the cytoplasm, and they contain 1 or multiple exons, with 2 or 3 exons derived from alternative splicing^27,30^. There are 3 hypothetical models explaining the biogenesis of ecircRNAs: lariat-driven circularization, intron-pairing-driven circularization, and RNA binding protein (RBP)-mediated circularization^31^. In the generation of ecircRNAs, partial RNA folding occurs during pre-mRNA transcription, and exons are skipped as the RNA is folded. These structural changes result in the formation of specific regions called lariat structures, in which initially non-adjacent exons, along with their introns, become close to each other. CircRNAs are then formed after the intron sequence is removed through splicing within the lariat structure. This model is defined as lariat-driven circularization. Reverse complementary sequences are present in introns on both sides of the pre-mRNA; therefore, the complementary pairing of introns on both sides mediates circRNA generation. This model is defined as intron-pairing-driven circularization. RBPs that function as transacting activators or inhibitors play significant roles in regulating circRNA production. The RNA editing enzyme adenosine deaminase 1 (ADAR1) antagonizes circRNA biogenesis by directly weakening inverted ALU repeats through A-to-I editing of RNA pairing flanking circularized exons, thereby decreasing the complementarity and stability of intron base pairing interactions^27,32,33^. DHX9, an abundant nuclear RNA helicase, is another ALU element target similar to ADAR1. Loss of DHX9 increases circRNA production through a process mediated by unwinding of RNA pairs flanking circularized exons^34^. In addition, several splicing factors can bind specific RNA motifs and subsequently regulate circRNA biogenesis. Quaking (QKI), a mesenchymal splicing factor, contributes to the regulation of circRNA production in epithelial-mesenchymal transition (EMT)^35^. The RNA-binding protein FUS controls back-splicing reactions leading to circRNA production in mouse embryonic stem cell-derived motor neurons^36^. EIciRNAs are composed of exons and retained introns, and are predominantly localized to the nucleus, where they promote the transcription of parental genes^28^. In the generation of ecircRNAs, introns that surround the exons are usually spliced out. However, in some cases, introns are retained and subsequently form EIciRNAs^28^. Although some lariat structures are generated by introns during splicing, most are degraded rapidly through debranching. Only some lariat structures containing essential nucleic acid sequences, such as a 7 nt GU-rich element adjacent to the 5′ splice site and an 11 nt C-rich element adjacent to the branch point site, are not debranched after splicing, thus forming ciRNAs^29^. The biogenesis of circRNAs is shown in **Figure 1**. CircRNAs can also be generated from lncRNAs, such as long intergenic non-protein-coding RNA p53-induced transcript (LINC-PINT), which have been shown to encode regulatory peptides in glioblastoma, thus revealing the abundance and complexity of RNA sources^37^.

## The function of circRNAs

### CircRNAs act as miRNA sponges or competing endogenous RNAs

According to the competing endogenous RNA (ceRNA) hypothesis, miRNAs act as gene expression regulatory elements that affect mRNA stability and translation at the post-transcriptional level through direct base pairing with target sites; miRNA activity can be affected by miRNA sponge transcripts^38,39^. CircRNAs that contain various types and numbers of miRNA binding sites can function as ceRNAs or miRNA sponges, thus inhibiting miRNAs and consequently regulating the expression of miRNA-related target gene by MREs^39,40^. The most widely known circRNA is ciRS-7 (circRNA sponge for miR-7), which contains more than 70 selectively conserved miRNA target sites and is highly associated with Argonaute (AGO) proteins in a miR-7-dependent manner^39^. In human and murine brain tissues, ciRS-7 acts as a molecular sponge for miR-7 and inhibits miRNA function, thus positively regulating miR-7 target genes. Kleaveland et al.^41^ have found a regulatory network centered on 4 ncRNAs—the lncRNA Cyrano, the circRNA ciRS-7, and 2 miRNAs, miR-671 and miR-7—in the mammalian brain. Loss of Cyrano increases miR-7 levels, thus causing cytoplasmic destruction of Cdr1as in neurons, partly through enhanced miR-671-directed slicing. CircSry, which is derived from the sex-determining region Y, serves as a miR-138 sponge in the mouse testis, thus providing early evidence that circRNAs can act as molecular sponges^12,39^. Another well-known circRNA, circHIPK3, acts as a sponge for 9 miRNAs, with 18 potential binding sites in multiple human tissues, including miR-124, miR-7, miR-4288, and miR-558^42–45^. Circ-ITCH inhibits bladder cancer progression by sponging miR-17/miR-224 and regulating p21 and PTEN expression^46^. Together, these findings suggest that the miRNA sponging effects of circRNAs may be a general phenomenon in cancer.

### CircRNAs regulate transcription and splicing

Although numerous studies support the roles of circRNAs as miRNA sponges, ElciRNAs and ciRNAs, which are primarily enriched in the nucleus, had been demonstrated to regulate gene expression in a transcriptional or post-transcriptional manner^28,29^. EIciRNAs, such as EIciPAIP2 and EIciEIF3J, are predominantly localized to the nucleus, where they interact with U1 small nuclear ribonucleoproteins and RNA pol II, and promote the transcription of parental genes^28^. Linear splicing and circRNA formation mutually regulate each other through competition for splice sites, in a manner that is tissue specific and conserved in animals^25^.

### CircRNAs bind proteins

Certain circRNAs interact with different proteins and form circRNA-protein complexes that regulate the subcellular localization of proteins, the activity of associated proteins, and the transcription of parental or related genes. Du et al.^47^ have demonstrated that circ-Foxo3 interacts with the senescence-related proteins ID1 and E2F1, and the stress-related proteins HIF1a and FAK, thus increasing cellular senescence through the suppression of anti-senescent and anti-stress roles. Another study has shown that the circ-Foxo3 interacts with the cell cycle proteins cyclin-dependent kinase 2 (CDK2) and cyclin-dependent kinase inhibitor 1 (p21), thereby hindering cell cycle progression *via* the formation of the circ-Foxo3-p21-CDK2 ternary complex^48^. In addition, circ-Foxo3 binds both p53 and MDM2 and subsequently promotes MDM2-induced p53 ubiquitination and represses MDM2-induced Foxo3 ubiquitination, thereby inducing cell apoptosis^49^. Abdelmohsen et al.^50^ have proposed that the extensive binding of circPABPN1 to HuR prevents HuR binding to PABPN1 mRNA and suppresses PABPN1 translation, thus providing an example of competition between a circRNA and its cognate mRNA for an RBP that affects translation. In another example, circACC1 affects cellular responses to metabolic stress and promotes the enzymatic activity of the AMPK holoenzyme by forming a ternary complex with the regulatory β and γ subunits^51^.

### CircRNAs can be translated

Early studies have reported that circRNAs, as non-coding RNAs, cannot be translated *via* cap-dependent mechanisms because of their lack of a 5′ cap and 3′ poly(A) tail^17^. However, increasing recent evidence indicates that circRNAs encode proteins. A circRNA database called circRNADb lists 16,328 circRNAs containing an open reading frame (ORF) longer than 100 amino acids, of which 7,170 have internal ribosome entry site (IRES) elements^52^. In addition, 46 circRNAs from 37 genes express proteins, according to mass spectrometry. Circ-ZNF609, which contains an ORF, can be translated into a protein in a splicing-dependent and cap-independent manner, thus providing an example of a protein-coding circRNA in eukaryotes^53^. Yang et al.^54^ have proposed that the spanning junction ORF in circ-FBXW7 driven by IRES encodes a novel 21-kDa protein, termed FBXW7-185aa, which decreases the half-life of c-Myc by antagonizing USP28-induced c-Myc stabilization, and inhibits proliferation and progression in glioblastoma. Another example is circ-SHPRH, which uses overlapping genetic codes to generate a UGA stop codon, thereby resulting in the translation of the 17 kDa SHPRH-146aa^55^. CircFBXW7, which acts as a sponge for miR-197-3p, encodes the FBXW7-185aa protein, which suppresses triple-negative breast cancer (TNBC) progression by upregulating FBXW7 expression. Zhang et al.^37^ have identified a peptide encoded by the circular LINC-PINT, which directly interacts with polymerase associated factor complex (PAF1c) and inhibits the transcriptional elongation of multiple oncogenes.

In addition to the IRESs and ORFs responsible for circRNA translation, N6-methyladenosine (m6A), the most abundant base modification of RNA, efficiently promotes protein translation from circRNAs in human cells^56^. m6A-driven translation requires the initiation factor eIF4G2 and the m6A reader YTHDF3; it is enhanced by the methyltransferases METTL3/14, inhibited by the demethylase FTO, and upregulated by heat shock. Although the translation of circRNAs has recently been identified, additional coding circRNAs and the mechanisms underlying their translation remain elusive.

### The study of circRNAs in BC

**Figure 2:**
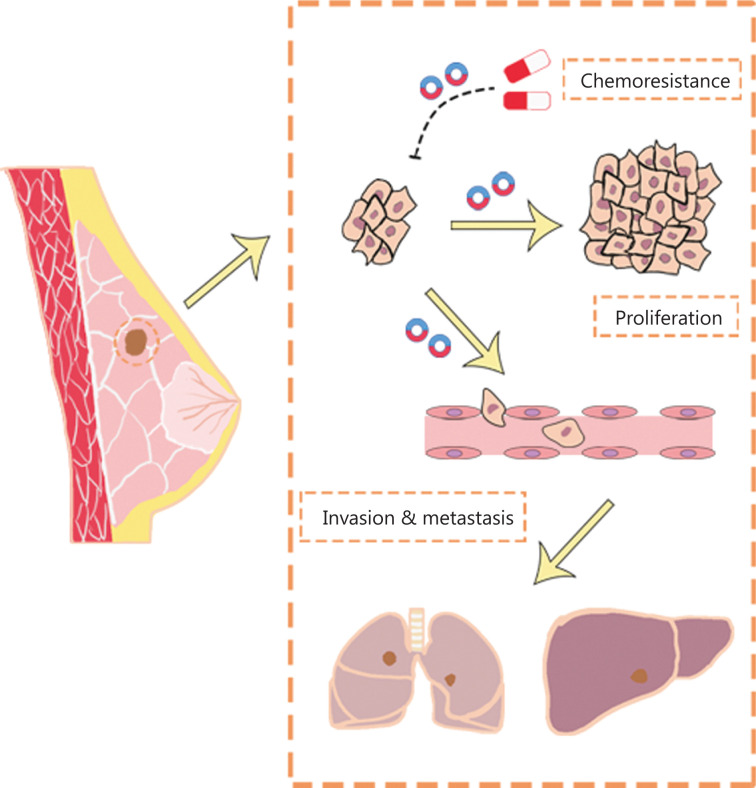
The function of circRNAs in BC. CircRNAs affect the proliferation, progression, and chemoresistance of BC.

**Figure 3:**
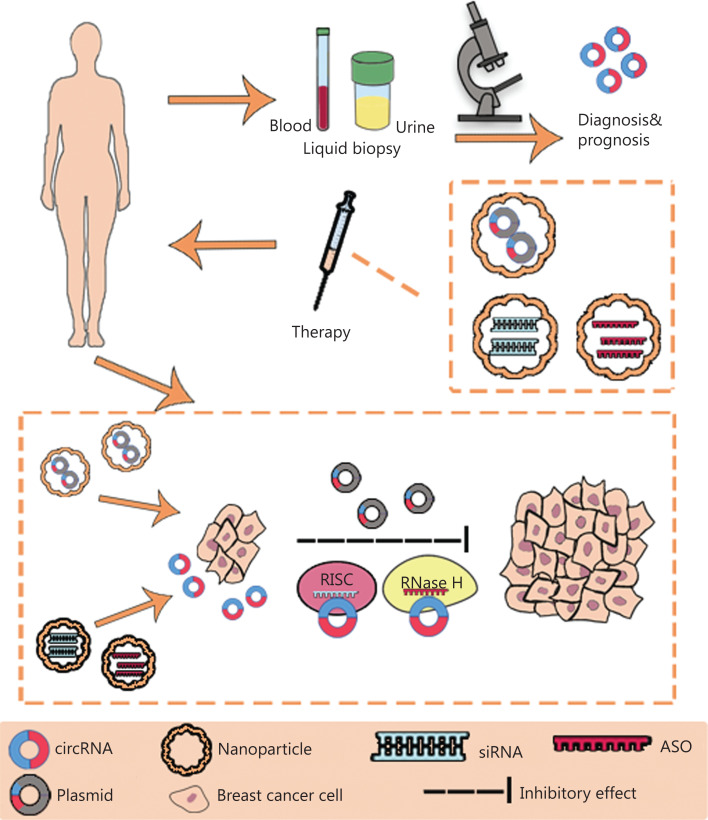
The clinical application of circRNAs in BC. CircRNAs have potential as novel diagnostic biomarkers and effective therapeutic targets. CircRNAs can be measured in liquid biopsy samples, such as the blood and urine, thus suggesting their potential as novel diagnostic and prognostic biomarkers in BC. Plasmids containing sequences encoding circRNAs, siRNAs and anti-sense oligonucleotides (ASO) can be delivered through nanoparticles *in vivo* and subsequently regulate the expression and function of circRNAs in BC. RISC, RNA-induced silencing complex; RNase H, ribonuclease H.

The development of high-throughput sequencing and circRNA microarrays has led to the identification of an increasing number of circRNAs with potential value in BC diagnosis, treatment, and prognostication. The functions (**Figure 2**) and clinical applications (**Figure 3**) of circRNAs in BC are described below.

### Expression of circRNAs in BC

Dysregulated expression of circRNAs in BC tissues compared with adjacent normal tissues has oncogenic or tumor-suppressive roles in the initiation and progression of BC. A study has identified 1,155 circRNAs including 715 upregulated and 440 downregulated circRNAs, that are aberrantly expressed in BC tissues compared with adjacent normal-appearing tissues^57^. Tang et al.^58^ have shown through circRNA microarray analysis that 1,705 circRNAs are aberrant in BC tissue. Yin et al.^59^ have detected 19 upregulated circRNAs and 22 downregulated circRNAs in plasma specimens from patients with BC. The number reported above may not represent the actual number of altered circRNAs in BC, and further studies are needed to identify additional dysregulated circRNAs. **Table 1** lists the dysregulated circRNAs in BC reported in the PubMed database.

**Table 1:** The expression and mechanisms of circRNAs in BC

Name	Expression	Function	Mechanism	Reference
circ-ABCB10	Up	Oncogene	miR-1271	^60^
circDENND4C	Up	Oncogene	HIF1α	^61^
circGFRA1	Up	Oncogene in TNBC	miR-34a/GFRA1	^62^
hsa_circ_0001982	Up	Oncogene	miR-143	^58^
circRNA_BARD1	Up	Oncogene	miR-3942-3p/BARD1	^63^
hsa_circ_0011946	Up	Oncogene	miR-26a/b-RFC3	^64^
circMYO9B	Up	Oncogene	miR-4316/FOXP4	^65^
circIRAK3	Up	Metastasis	miR-3607/FOXC1	^66^
hsa_circ_0008039	Up	Oncogene	miR-432-5p/E2F3	^67^
circANKS1B	Up	Metastasis	miR-148a-3p/152-3p-USF1	^68^
hsa_circ_0007534	Up	Oncogene	miR-593/MUC19	^69^
ciRS-7	Up	Oncogene in TNBC	miR-1299/MMPs	^70^
circRNA‑MTO1	Up	Monastrol resistance	TRAF4/Eg5	^71^
circ-DNMT1	Up	Oncogene	Activation of autophagy	^72^
circEPSTI1	Up	Oncogene in TNBC	miR-4753/6809-BCL11A	^73^
FECR1	Up	Metastasis	TET1 and DNMT1	^74^
circ-UBAP2	Up	Oncogene in TNBC	miR-661/MTA1	^75^
circZNF609	Up	Oncogene	miR-145-5p/p70S6K1	^76^
hsa_circ_0072995	Up	Oncogene	miR-30c-2-3p	^77^
hsa_circ_0052112	Up	Oncogene	miR-125a-5p	^78^
hsa_circ_0006528	Up	Oncogene and adriamycin resistance	miR-7-5p/MAPK/ERK pathway	^79^
hsa_circ_001569	Up	Oncogene	PI3K-AKT pathway	^80^
circAGFG1	Up	Oncogene in TNBC	miR-195-5p/CCNE1	^81^
hsa_circ_001783	Up	Oncogene	miR-200c-3p	^82^
circKIF4A	Up	Oncogene in TNBC	miR-375/KIF4A	^83^
CDR1as	Up	5-fluorouracil resistance	miR-7/CCNE1	^84^
circ_0103552	Up	Oncogene	miR-1236	^85^
hsa_circ_0136666	Up	Oncogene	miR-1299/CDK6	^86^
circRNA‑CER	Up	Oncogene	miR‑136/MMP13	^87^
hsa_circ_0004771	Up	Oncogene	miR-653/ZEB2	^88^
circRNA_069718	Up	Oncogene in TNBC	Wnt/β-catenin pathway	^89^
circANKRD12	Up	Oncogene	Cell cycle	^90^
circRNA_100876	Up	Oncogene	miR-361-3p	^91^
circPLK1	Up	Oncogene in TNBC	miR-296-5p/PLK1	^92^
circ-UBE2D2	Up	Oncogene	miR-1236/1287	^93^
hsa_circ_21439	Up	Metastases	ceRNA	^94^
circAMOTL1	Up	PTX resistance	AKT pathway	^95^
circDENND4C	Up	Oncogene	miR-200b/200c	^96^
circ_0067934	Up	Oncogene	Mcl-1	^97^
circ-TFCP2L1	Up	Oncogene in TNBC	miR-7/PAK1	^98^
circFBXL5	Up	Oncogene	miR-660/SRSF6	^99^
circ_0001667	Up	Oncogene	miR-125a-5p/TAZ	^100^
circACAP2	Up	Oncogene	miR-29a/b-3p-COL5A1	^101^
circVAPA	Up	Metastases	miR-130a-5p	^102^
hsa_circ_002178	Up	Oncogene	miR-328-3p/COL1A1	^103^
circ-TFF1	Up	Oncogene	miR-326/TFF1	^104^
circ_0007255	Up	Oncogene	miR-335-5p/SIX2	^105^
hsa_circ_0008039	Up	Oncogene	miR-515-5p/CBX4	^106^
circRNF20	Up	Oncogene	miR-487a/HIF-1α/HK2	^107^
hsa_circ_0091074	Up	Oncogene in TNBC	miR‑1297/TAZ/TEAD4	^108^
circSKA3	Up	Oncogene	Complex with Tks5 and integrin β1	^109^
circCDYL	Up	Oncogene	miR-1275-ATG7/ULK1	^110^
circ_103809	Up	Oncogene	PI3K/AKT	^111^
circABCB10	Up	Oncogene and PTX resistance	let-7a-5p/DUSP7	^112^
circABCB10	Up	Oncogene and radioresistance	miR-223-3p/PFN2	^113^
circGNB1	Up	Oncogene in TNBC	miR-141-5p/IGF1R	^114^
circSEPT9	Up	Oncogene in TNBC	miR-637/LIF	^115^
circHMCU	Up	Oncogene	let-7 family	^116^
circKIF4A	Up	Oncogene	miR-152/ZEB1	^117^
circRAD18	Up	Oncogene	miR-613/HK2	^118^
circRAD18	Up	Oncogene in TNBC	miR-208a/3164-IGF1/FGF2	^119^
circ-RNF111	Up	PTX resistance	miR-140-5p/E2F3	^120^
hsa_circ_0000515	Up	Oncogene	miRNA-296-5p/CXCL10	^121^
hsa_circ_0131242	Up	Oncogene in TNBC	hsa-miR-2682	^122^
circIFI30	Up	Oncogene in TNBC	miR-520b-3p/CD44	^123^
circ_DCAF6	Up	Oncogene	miR-616-3p/GLI1/hedgehog pathway	^124^
circEIF3M	Up	Oncogene in TNBC	miR-33a/cyclin D1	^125^
circ-ZEB1	Up	Oncogene in TNBC	miR-448/eEF2 K	^126^
hsa_circRPPH1_015	Up	Oncogene	miRNA-326/ELK1	^127^
circMMP11	Up	Oncogene	miR-1204/MMP11	^128^
circ-Foxo3	Down	TS	p53 and MDM2	^49^
circRNA-000911	Down	TS	miR‑449a	^129^
circ-Ccnb1	Down	TS	p53 mutation	^130^
circ-ITCH	Down	TS in TNBC	miR-214/17-Wnt/β-catenin pathway	^131^
circASS1	Down	Inhibition of metastasis	miR-4443/ASS1	^132^
hsa_circ_0072309	Down	TS	miR-492	^133^
circYap	Down	TS	Suppression of translation	^134^
circTADA2As	Down	TS in TNBC	miR-203a-3p/SOCS3	^135^
circRNA_0025202	Down	TS and tamoxifen resistance	miR-182-5p/FOXO3a	^136^
circBMPR2	Down	TS and tamoxifen resistance	miR-553/USP4	^137^
circFBXW7	Down	TS in TNBC	miR-197-3p/FBXW7, and encoded 185-aa protein	^138^
circKDM4C	Down	TS and doxorubicin resistance	miR-548p/PBLD	^139^
circAHNAK1	Down	TS in TNBC	miR-421/RASA1	^140^
hsa_circ_0000376	Down	TS	miR-1285-3p/SMURF1-BTRC-TP53	^141^
hsa_circ_0068033	Down	TS	miR-659	^142^
circ-LARP4	Down	Doxorubicin resistance	/	^143^
circSCYL2	Down	Inhibition of metastasis	EMT	^144^
circEHMT1	Down	TS	miR-1233-3p/KLF4/MMP2	^145^
circNFIC	Down	TS	miR-658/UPK1A	^146^
circRNA_103809	Down	TS	miR-532-3p	^147^
circ-1073	Down	TS	Interaction with HuR	^148^
circDDX17	Down	TS	miR-605/CDK1 and p21	^149^

TS, tumor suppressor; Up, upregulation; Down, downregulation; TNBC, triple-negative breast cancer.

### CircRNAs affect the proliferation and progression of BC through a ceRNA mechanism

Increasing evidence supports the association between the circRNA-miRNA-mRNA network and tumorigenesis. CircRNAs function as miRNAs sponges that regulate the proliferation, metastasis, and invasion of cancers, including BC. For example, circKIF4A, which is associated with poorer outcomes in TNBC, exerts its regulatory functions in TNBC through regulating the expression of KIF4A by sponging miR-375^83^. The activity of circSEPT9, mediated by E2F1 and EIF4A3, promotes carcinogenesis and TNBC development through the circSEPT9/miR-637/LIF axis^115^. Liang et al.^110^ have demonstrated that circCDYL promotes autophagy by sponging miR-1275 and upregulates the expression of the autophagy-associated gene ATG7 and ULK1. CircHMCU is upregulated in human BC and promotes the proliferation, migration, and invasion of BC cells by affecting the G1 phase cell cycle checkpoint and the EMT pathway. In addition, circHMCU binds miRNA let-7 family members, thus increasing the expression of let-7 family target genes such as MYC, HMGA2, and CCND1^116^. Certain circRNAs, such as circIRAK3^66^, circANKS1B^68^, and ciRS-7^70^, promote BC cell migration, invasion, and metastasis, but have no effect on the proliferation and growth of BC. CircIRAK3 is upregulated in metastatic BC cells and exerts regulatory roles in BC metastasis *via* the circIRAK3/miR-3607/FOXC1 signaling axis^66^. Zeng et al.^68^ have characterized a novel circRNA, circANKS1B, that is upregulated in TNBC and promotes BC invasion and metastasis by inducing EMT. Mechanistically, circANKS1B sponges miR-148a-3p and miR-152-3p, thereby upregulating expression of the transcription factor USF1, which in turn upregulates TGF-β1 expression, activates TGF-β1/Smad signaling, and promotes EMT. Together, these findings suggest that circRNA-miRNA-mRNA interaction networks play vital roles in BC proliferation and progression.

### CircRNAs affect the proliferation and progression of BC through cancer-associated signaling pathways

Multiple cancer-associated signaling pathways are involved in the proliferation and progression of BC. In addition to the ceRNA mechanism, substantial evidence indicates that circRNAs play key roles in the proliferation, migration, invasion, and metastasis of BC by regulating the expression of target genes directly or by interacting with mRNAs or proteins associated with cancer-related signaling pathways. Mutant p53 suppresses cancer progression and malignancy^150^. Circ-Ccnb1, which is downregulated in BC, binds H2AX and wild-type p53, thus preventing the induction of cell death; however, in p53 mutant cells, circ-Ccnb1 forms a complex with H2AX and Bclaf1 and induces cancer cell death and inhibition of tumor progression^151^. Du et al.^72^ have shown that ectopically expressed circ-Dnmt1 interacts with both p53 and AUF1, and promotes the nuclear translocation of both proteins, thereby increasing the proliferation and survival of BC cells through stimulating cellular autophagy. The nuclear translocation of p53 induces cellular autophagy, whereas the nuclear translocation of AUF1 increases Dnmt1 mRNA stability and translation, thus inhibiting p53 transcription and promoting autophagy. Chen et al.^74^ have identified a novel FLI1 circRNA called FECR1, which regulates metastasis of BC cells by coordinating the DNA methylation and demethylation of target genes involved in tumor growth. FECR1 not only interacts with the FLI1 promoter in cis and recruits TET1 demethylase, but also binds the Dnmt1 promoter and downregulates Dnmt1 in trans. Wu et al.^134^ have presented evidence that circYap binds Yap mRNA and the translation initiation associated proteins eIF4G and PABP, thus suppressing Yap translation initiation and delaying tumor progression. CircSKA3 promotes tumor progression by forming a complex with Tks5 and integrin β1, thereby inducing invadopodium formation^109^. CircRNA_069718 promotes TNBC progression by activating the Wnt/β-catenin pathway^89^. Hsa_circ_001569 and hsa_circ_103809 promote BC progression by regulating the PI3K/AKT signaling pathway^80,111^. Despite extensive research, many of the cancer-associated signaling pathways mediating the regulatory functions of circRNAs in BC progression remain unknown. The identification of other circRNAs involved in cancer-associated signaling pathways may improve understanding of the molecular mechanisms underlying BC.

### CircRNAs function as potential novel biomarkers for BC diagnosis and prognostication

The limitations of mammography, breast ultrasound, and histopathology in the early diagnosis of BC underscore the need to identify novel biomarkers for early BC detection and prognostication. The main characteristics of circRNAs are their abundance, stability, conservatism, location, and specificity. Because of these features, circRNAs show promise as potential noninvasive biomarkers for diagnosis and prognostication^152^. CircRNAs can be detected in body fluids (such as blood, plasma, serum, and exosomes) from patients with BC and are associated with clinicopathological characteristics, survival time, and prognosis. Wang et al.^153^ have identified 1,147 and 1,195 circRNAs that are dysregulated in exosomes from patients with metastatic and localized BC, respectively. CircCNOT2 is detectable in cell-free plasma RNA and is a predictor of progression-free survival time in patients with advanced breast cancer receiving aromatase inhibitor therapy^154^. Li et al.^155^ have shown that hsa_circ_0069094, hsa_circ_0079876, hsa_circ_0017650, and hsa_circ_0017526 are upregulated in the plasma of patients with BC and are significantly associated with tumor volume, TNM stage, and lymph node infiltration. Patients with BC with higher circCDYL levels in the serum or tumor tissues have a higher tumor burden, shorter survival, and poorer clinical response to therapy^110^. These examples are only a fraction of the instances in which circRNAs have been shown to have diagnostic and prognostic potential in BC. Therefore, the clinical application of circRNAs as biomarkers for diagnosis and prognostication must be further studied in the future.

### CircRNAs function as potential novel therapeutic targets in BC

Many circRNAs are involved in the growth, progression, and drug resistance of BC, thus suggesting that circRNAs have great potential as novel therapeutic targets. Some circRNAs are upregulated in BC tissues and function as oncogenes through a ceRNA mechanism or cancer-associated signal pathways. Many circRNAs have various binding sites for specific miRNAs, and are thus effective miRNA inhibitors through the circRNA-miRNA-mRNA network. Advances in technologies such as siRNA-based therapy^156^, anti-sense oligonucleotide therapy^157^, and the CRISPR/Cas system^158^, among others, may enable the downregulation or suppression of oncogenic circRNAs in the future. In addition, epigenetic modifications of RNA, including m6A, play significant roles in regulating the biogenesis and metabolism of circRNAs. A recent study has found that a subset of m6A-containing circRNAs interacts with YTHDF2 in an HRSP12-dependent manner and is selectively downregulated by the RNase P/MRP, thus representing a potential novel way to degrade oncogenic circRNAs^159^. The study has also indicated that the permuted intron-exon method may be a promising method for development of new drugs associated with circRNAs^160^.

Recent studies have shown that some circRNAs are downregulated in BC tissues and function as tumor suppressors. CircTADA2As has been found to be significantly decreased in a large cohort of patients with BC, and to suppress BC progression and metastasis by targeting the miR-203a-3p/SOCS3 axis^135^. The downregulation of circTADA2As is associated with poor patient survival in TNBC. Ye et al.^138^ have demonstrated that circFBXW7 sponges miR-197-3p and encodes the FBXW7-185aa protein, thus suppressing TNBC progression by upregulating FBXW7 expression. Circ-1073 functions as a tumor suppressor in BC. Injection of nanoparticles containing a plasmid encoding circ-1073 has been shown to inhibit the growth of xenograft tumors; consequently, circRNAs in the plasma may contribute to the suppression of BC *in vivo*^148^. Lin et al.^161^ have recently developed a TV-circRGPD6 nanoparticle that selectively expresses circRGPD6 in metastatic breast cancer stem cells and eradicates BC metastasis. These findings indicate that the development of nanotechnology, including synthetic tumor inhibitors, may lead to the application of circRNAs in BC treatment.

Chemotherapy is an effective strategy for the clinical treatment of BC, and chemoresistance is a major cause of treatment failure and poor prognosis in patients with BC. Therefore, clarifying the molecular pathways associated with chemoresistance, increasing the chemosensitivity of cancer cells, and predicting the efficacy of chemotherapeutic agents in individual patients are vital for the clinical management of BC. CircRNAs are involved in the pathogenesis of BC associated with chemotherapy resistance. Gao et al.^162^ have found that circ_0006528 is upregulated in adriamycin-resistant BC cells and tissues and plays a role in BC chemoresistance *via* the circ0006528-miR-7–5p-Raf1 axis. The circRNA CDR1as decreases the chemosensitivity of 5-fluorouracil resistant BC cells by inhibiting miR-7, thus regulating CCNE1^84^. Hsa_circ_0025202, which is downregulated in BC, functions as a miR-182-5p sponge and relieves the suppression of FOXO3a, thereby increasing cell apoptosis and sensitivity to tamoxifen^136^. Liang et al.^137^ have demonstrated that circBMPR2 inhibits the progression and tamoxifen resistance of BC *via* the circBMPR2/miR-553/USP4 axis. CircBMPR2 knockdown promotes tamoxifen resistance in BC cells by inhibiting tamoxifen-induced apoptosis, whereas circBMPR2 overexpression decreases tamoxifen resistance. CircKDM4C suppresses tumor progression and attenuates doxorubicin resistance by regulating the miR-548p/PBLD axis in BC^139^. Paclitaxel (PTX) has been approved for use alone or in combination with other drugs against BC^163^. Cells overexpressing circAMOTL1 show increased resistance to PTX, but not epirubicin and cyclophosphamide, in BC. Mechanically, circAMOTL1 suppresses cell apoptosis and promotes cell survival by activating AKT, and phosphorylated AKT upregulates the anti-apoptotic gene BCL2 and inhibits the proapoptotic genes BAX and BAK^95^. Circ-ABCB10 depletion promotes PTX sensitivity and apoptosis, and suppresses the invasion and autophagy of PTX-resistant BC cells *via* the let-7a-5p/DUSP7 axis^112^. Circ-RNF111, which is upregulated in PTX-resistant BC tissues, decreases PTX resistance in BC by upregulating E2F3 *via* sponging miR-140-5p^120^. The roles of circRNAs in chemotherapy resistance and the clinical application of circRNAs to overcome chemotherapy resistance require further exploration.

## Conclusions

Extensive studies have confirmed that circRNAs play key roles in the initiation and progression of BC, thus suggesting their potential as novel diagnostic biomarkers and effective therapeutic targets. However, the mechanisms underlying the association between circRNAs and BC remain elusive, and the clinical application of circRNAs has consequently been limited. Elucidation of the molecular mechanisms underlying the role of circRNAs in BC is necessary.
